# Bilateral internal jugular vein (BIJV) sampling during surgery for primary hyperparathyroidism (PHPT) – scoping review of evidence and search for an optimal definition for lateralisation

**DOI:** 10.1007/s00423-025-03957-5

**Published:** 2026-01-02

**Authors:** Varun Prakash, Nithilan Kamalakkannan, Saba P. Balasubramanian

**Affiliations:** 1 University of Newcastle Medical School, Newcastle upon Tyne, UK; 2 Medical school, University of Sheffield, Sheffield, UK; 3 Sheffield Teaching Hospitals NHS Foundation Trust, Sheffield, UK

**Keywords:** Primary hyperparathyroidism, Parathyroid hormone, Surgery, BIJVS

## Abstract

**Purpose:**

Accurate localisation of hyperfunctioning parathyroid glands is crucial for successful parathyroid surgery. In patients with inconclusive imaging, intraoperative bilateral internal jugular venous sampling (BIJVS) has been reported; but its utility remains unclear. The purpose of the review is to evaluate published techniques and reported effectiveness of BIJVS in parathyroid surgery.

**Methods:**

PubMed, Ovid and Cochrane databases were searched for articles on intraoperative BIJVS in parathyroid surgery. All original English language human studies reporting on lateralisation rates, diagnostic accuracy or cure rates following use of intraoperative BIJVS were included. Exclusion criteria included case reports, reviews, IJV sampling in non-parathyroid pathology and IJV sampling for confirming cure. Data on patient numbers, definitions used for lateralisation and correlation with clinical outcomes were extracted by one reviewer and cross-checked by a second reviewer. The review was prospectively registered on the Open Science Framework (OSF; DOI: 10.17605/OSF.IO/TSQA6).

**Results:**

Of 753 screened, 12 studies including 502 patients where BIJVS was performed were included. Lateralisation definitions were reported in 7 studies. Among studies with relevant data, lateralisation gradient was defined as ranging from 5 to 20% and lateralisation rates varied from 51 to 100%. The positive and negative predictive values ranged from 76 to 100% (6 studies) and 0–53% respectively (3 studies). Reported cure rates following BIJVS guided surgery were high (> 98%), but the definition for cure was only reported in 8 studies.

**Conclusions:**

BIJVS can aid localisation in parathyroid surgery. A significant lateralisation gradient may permit unilateral surgery, but a lack of gradient does not imply bilateral disease. However, the absence of a standard definition for lateralisation and inconsistent reporting limits widespread adoption of this technique.

## Introduction

Primary hyperparathyroidism is the most common cause of hypercalcaemia in the outpatient setting and typically results from a solitary parathyroid adenoma in 80–85% of cases [1]. The morbidity of PHPT includes osteoporosis, nephrolithiasis, nephrocalcinosis, renal impairment, neurocognitive problems, impaired quality of life, and increased risk of cardiovascular disease [2]. The definitive treatment for PHPT is a parathyroidectomy, offering a high cure rate of 95% and above [3].

For patients with PHPT undergoing surgery, accurate preoperative localisation of the hyperfunctioning parathyroid gland(s) is critical for successful surgery. Preoperative imaging modalities such as ultrasound and MIBI scan are commonly used to localise the adenoma [4]. Other imaging modalities such as 4D-CT scan and choline PET scan are also used but are commonly reserved for patients with failed primary surgery or recurrent disease [5]. However, these imaging techniques do not always provide clear results, especially in cases where multiglandular disease is present or when re-operative surgery is performed. The results of preoperative localisation studies significantly influence the surgical approach. While unilateral or targeted exploration is often adequate for clearly localised disease where a single adenoma is most likely, bilateral neck exploration remains necessary for cases with unclear or discordant imaging results [6].

Intraoperative parathyroid hormone monitoring is becoming increasingly used in parathyroid surgery. It traditionally works by measuring the drop in parathyroid hormone (PTH) levels following the excision of hyperfunctioning parathyroid gland(s), confirming that all hyperfunctioning tissue has been removed. A significant reduction in PTH levels (by around 50%) after gland excision is indicative of a biochemical cure [7]. However, there are limitations to IOPTH, including cost, potential for false negatives or false positives, and extended operative times [8].

BIJVS is another technique used before or during surgery to further aid localisation of hyperfunctioning parathyroid glands. By measuring PTH levels from both the left and right internal jugular veins, a significant difference in PTH levels can help guide surgeons to the side of the abnormal parathyroid gland [9]. The technique may be particularly useful in cases where preoperative imaging is inconclusive. However, the method’s efficacy and its role in modern parathyroid surgery is debatable due to no clear consensus on the optimal way to define lateralisation and interpret the results provided [10]. Variability in protocols, particularly regarding lateralisation thresholds, has contributed to inconsistent uptake and heterogeneous reporting in the literature. Despite these reports, the role of BIJVS remains uncertain, largely due to heterogeneity in techniques, thresholds for lateralisation, and outcome reporting. This variability has limited standardisation and widespread adoption, highlighting the need for a scoping review.

While some studies report high sensitivity and specificity [9], others suggest limited benefits, especially in re-operative surgery or when preoperative imaging is clear [11]. Given the uncertainties associated with BIJVS, this scoping review aims to collate existing evidence, assess the accuracy of BIJVS in different surgical contexts (primary and secondary hyperparathyroidism; first-time and re-do surgery), and explore how lateralisation is defined and used in the literature.

## Materials and methods

### Search strategy

A literature search was performed using the PubMed, Cochrane library and Ovid databases on the 19th of April 2025. The search strategy was designed to identify studies evaluating the use of BIJVS for localisation or lateralisation in the surgical management of primary hyperparathyroidism. The following search string was used across all databases: (parathyroid hormone OR PTH) AND (surgery OR operative OR intraoperative) AND (internal jugular vein OR jugular OR IJV). Following the initial PubMed search, articles in non-English language and non-human studies were excluded. In the Ovid database, the search was restricted to Ovid Medline^®^ and Epub ahead of Print, In-process, In-data-Review and Other non-indexed citations, daily and versions < 1946 to April 18, 2025. In Cochrane, no filters were applied.

### Inclusion and exclusion criteria

Studies were eligible for inclusion if they were original research articles involving human subjects undergoing surgery for primary hyperparathyroidism, in which BIJVS was used for disease localisation or lateralisation during surgery. Studies were required to report outcomes such as surgical cure rate, localisation accuracy, concordance with imaging or diagnostic performance. Only studies with full-text available in English were considered.

Studies were excluded if they were case reports, narrative reviews, editorials, or conference abstracts without full data; if they focused on conditions other than parathyroid disease; or if IJVS was performed solely for biochemical confirmation of PTH drop without any intent to localise or lateralise disease. Studies were also excluded if BIJVS was conducted entirely in the preoperative setting.

### Screening and study selection process

Titles and abstracts were screened collaboratively by two reviewers. Screening was not performed independently. Data extraction was carried out by a single reviewer using a structured extraction form. A second reviewer subsequently reviewed the extracted data for completeness and accuracy. Discrepancies were resolved through discussion. No formal critical appraisal or risk of bias assessment was performed, in keeping with scoping review methodology as the aim was to map existing evidence rather than exclude studies based on methodological quality. No authors of any of the included manuscripts were contacted to obtain additional information.

The included studies comprised mainly observational case series and cohort studies, with one randomized controlled trial (Barczynski et al., 2009) directly comparing surgery with and without BIJVS. This RCT provides higher-level evidence within an otherwise predominantly observational dataset.

### Data extraction procedures

Extracted data included study design, country, number of centres, population characteristics, disease type (primary hyperparathyroidism), whether surgery was first-time or redo, preoperative imaging modalities and results, timing and technique of BIJVS, definition of surgical cure, number of patients who underwent or did not undergo BIJVS, cure rates stratified by BIJVS status, the definition and outcomes of lateralisation, and diagnostic metrics such as positive and negative predictive values. No assumptions were made for missing or ambiguous data unless the information could be clearly inferred from the study text. Data were summarized descriptively using narrative synthesis, with study-level characteristics tabulated and diagnostic outcomes presented as ranges. Where possible, cure rates and predictive values were reported as stated in the primary studies.

### Registration details

The review has been registered with the Open Science Framework (10.17605/OSF.IO/TSQA6*).* The reporting has been done in accordance with the PRISMA-SCR (PRISMA extension for scoping reviews) guidance [12]. PROSPERO registration was not applicable, as scoping reviews are not currently accepted.

### Risk of bias assessment and certainty of evidence

No formal risk-of-bias assessment was performed. The studies included in this review were highly heterogeneous in design ranging from small observational case series to one randomized controlled trial and many were not primarily conducted to evaluate BIJVS as a technique. Because the purpose of this scoping review was to map and describe the existing evidence rather than to evaluate the effectiveness of an intervention, the use of formal appraisal tools such as ROBINS-I or GRADE was not considered appropriate.

## Results

**Fig. 1:**
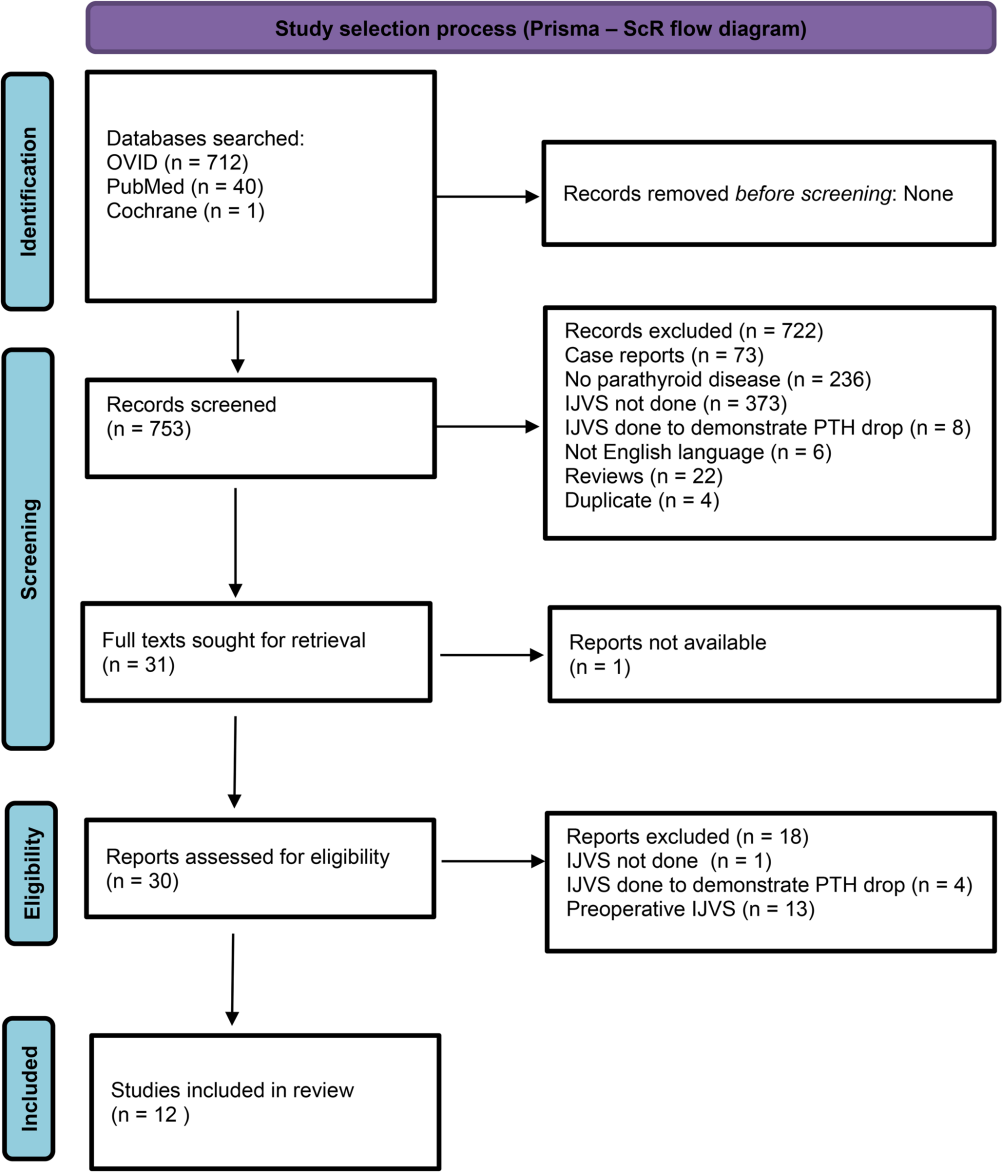
PRISMA flow diagram detailing process of identification, screening and inclusion of studies in this review. Figure legends: 1. Prisma flow diagram showing process of identification, screening and inclusion of studies in this scoping review. Note: IJVS – internal jugular vein sampling

Figure 1 shows the PRISMA flow diagram describing the screening and selection of studies that were included in this review. Twelve studies [8, 9, 13–22] involving a total of 2,490 patients with parathyroid disease undergoing surgery were included for analyses. Of these patients, 502 underwent BIJVS. All studies except one included patients with primary hyperparathyroidism (PHPT); the remaining study included a mixed cohort, i.e. some patients also had secondary hyperparathyroidism [9]. Most studies were observational in design (*n* = 11), while one was a randomised controlled trial [15]. Of the observational studies, 5 were single arm studies [9, 13, 19–21]. All were single-centre studies with varying sample sizes (ranging from 20 to 1133 patients). No critical appraisal of included studies was performed, consistent with the scoping nature of this review. Tables 1 and 2 summarises the results of these studies describing the numbers in different groups and reported cure rates.

**Table 1:** Characteristics of studies included in the review

Study	Country	Design	Arms	Population	Pre-op imaging results
Maceri et al. [13]	USA	Observational	1	56	Mixed
Alvarado et al. [14]	Australia	Observational	3	90	Mixed
Carneiro-Pla et al. [8]	USA	Observational	2	72	Scan negative
Barczynski et al. [15]	Poland	Randomized controlled trial	2	78	Scan negative
Sharma et al. [16]	USA	Observational	2	1133	Unclear
Ito et al. [9]	USA	Observational	1	168	Mixed
Lee et al. [17]	USA	Observational	2	466	Scan negative
Solorzano et al. [18]	USA	Observational	2	180	Scan negative
Taylor et al. [19]	UK	Observational	1	23	Mixed
Ribeiro et al. [20]	Brazil	Observational	1	29	Mixed
Bergenfelz et al. [21]	Sweden	Observational	1	20	Unclear
Pattou et al. [22]	France	Observational	3	175	NA

**Table 2:** Outcomes relevant to BIJVS as reported in included studies

Study	Lateralisation Definition	Lateralised (%)	Positive predictive Value	Negative Predictive Value	Cure Rate %
Maceri et al. [13]	NA	NA	NA	NA	NA
Alvarado et al. [14]	> 10% PTH difference between sides	34 (57%)	76%	12%	NA
Carneiro-Pla et al. [8]	> 10% difference in PTH between sides	10 (83%)	90%	0	100%
Barczynski et al. [15]	≥ 10% difference in PTH between sides	20 (51%)	85%	53%	100%
Sharma et al. [16]	NA	17 (77%)	NA	NA	NA
Ito et al. [9]	> 5% difference in PTH between sides	104 (69%)	86%	NA	98%
Lee et al. [17]	NA	3 (100%)	100%	0 (0%)	100%
Solorzano et al. [18]	NA	NA	NA	NA	NA
Taylor et al. [19]	NA	NA	NA	NA	100%
Ribeiro et al. [20]	> 20% difference in PTH between sides	22 (76%)	NA	NA	NA
Bergenfelz et al. [21]	Involved a formula incorporating comparison with peripheral vein PTH and assay variability	13 (65%)	92%	NA	NA
Pattou et al. [22]	> 20% difference compared to Peripheral vein PTH	NA	NA	NA	100%

Three studies only included first-time surgeries [14, 15, 21]; 1 study involved only re-operative (redo) surgeries [16]; and 3 studies included a mixed cohort of both first-time and redo surgeries [8, 18, 19]. Five studies did not explicitly specify the type of surgery performed [9, 13, 17, 20, 22].

Pre-operative imaging was performed in all except one study [22]. Imaging modalities typically included ultrasound, sestamibi scans, and CT imaging, although one study [21] did not specify which modality was used. The results from pre-operative imaging varied considerably: four studies only included patients with negative pre-operative scan results [8, 15, 17, 18], while another five included patients with both positive and negative pre-operative imaging findings [9, 13, 14, 19, 20] and one study did provide clearly what the pre-operative imaging results were [16].

In terms of timing, BIJVS was only performed intraoperatively in 11 studies. In one study [13], sampling was done before surgery in all patients, but this study was included as intraoperative sampling was also done in a small cohort of 12 patients. Five studies [9, 14, 15, 19, 21] performed BIJVS at the start of surgery after anaesthesia induction but before skin incision. Seven studies [8, 13, 16–18, 20, 22] performed BIJVS after incision. None of the included studies compared timing strategies on the accuracy or interpretability of the sampling results.

Only one study [15] directly compared outcomes in patients undergoing surgery with and without BIJVS as part of a randomised controlled trial. Baseline demographic and biochemical characteristics were comparable between groups. Cure rates were 100% in both arms at 6-month follow up. BIJVS significantly improved intraoperative localisation: sensitivity and positive predictive value were 65.4% and 85% with BIJVS compared to 30.8% and 44.4% with ultrasound alone. BIJVS reduced false-positive and false-negative rates by around half and increased the proportion of patients eligible for minimally invasive video-assisted parathyroidectomy (MIVAP) from 20.5% to 43.6%. These findings indicate that although overall cure rates were equivalent, BIJVS enhanced the accuracy of intraoperative decision making and enabled more patients to proceed with less invasive procedures.

Lateralisation criteria were explicitly defined in seven studies. Of these, five used a percentage difference in PTH levels between the two internal jugular veins, with thresholds ranging from 5% to 20% [8, 9, 14, 15, 20]. One study compared PTH levels in the jugular vein with peripheral vein PTH and employed a formula incorporating assay variability (1.96 × √2 × 0.07 × peripheral PTH - where 1.96 ensures 95% confidence, √2 accounts for variability in both jugular and peripheral samples, and 0.07 represents the assay’s 7% margin of error). If there was bilateral neck gradient or no gradient compared to peripheral veins, then the PTH values in the neck were then compared [21]. Another used a jugular-to-peripheral PTH ratio > 1.2 as the threshold for lateralisation [22].The remaining five studies [13, 16–19] did not state a clear definition for lateralisation, despite reporting lateralisation results.

Across the 8 studies [8, 9, 14–17, 20, 21] reporting lateralisation outcomes, the proportion of patients lateralised ranged from 51.3% to 100%. Six studies reported the positive predictive value (PPV) and 4 studies reported the negative predictive value (NPV), with the PPV ranging from 76.5% to 100% [8, 9, 14, 15, 17] and the NPV ranging from 0% to 52.6% [8, 14, 15, 17].

Cure rates following BIJVS-guided surgery were consistently high across included studies. Three studies provided a clearly defined cure based on postoperative biochemical criteria [9, 13, 14], while five others included relevant follow-up data (such as calcium or PTH levels) without explicitly defining ‘cure’ [8, 15, 16, 19, 22]. The definitions of cure from the three studies included a greater than 50% drop in pre-incision hormone levels at 5–10 min post-gland excision [13], normalization of serum calcium levels (< 2.62 mmol/L) at a 3-month follow-up [14], or maintenance of serum calcium levels below 10.2 mg/dL for at least 6 months after surgery [9]. Among those that did define cure clearly, reported rates ranged from 98% to 100%.

Overall, BIJVS was most consistently reported as an adjunct for localisation in patients with inconclusive imaging, with high positive predictive values and cure rates when lateralisation was achieved but less reliable when there was no lateralisation.

## Discussion

This scoping review synthesises evidence from twelve studies evaluating the role of BIJVS in the surgical management of hyperparathyroidism, with a focus on its utility in lateralizing hyperfunctioning parathyroid tissue. Across 2,490 patients, 507 underwent BIJVS, reflecting its selective use, often in complex clinical cases.

Lateralisation was explicitly defined in just over half of the included studies, with most using a percentage threshold for inter-jugular PTH gradients, typically between 5% and 20%. However, two studies adopted alternative thresholds, such as jugular-to-peripheral PTH ratios as well as give incorporating the effect of assay variability. The variability in definitions highlights a lack of standardisation in the use of BIJVS, which may impact comparability between studies and reproducibility in clinical practice.

Lateralisation success rates varied considerably, ranging from 51% to 100%. When lateralised, the PPVs were generally high (76–100%). Notably data on the NPVs were less frequently reported and showed greater variability ranging from (0%-53%). This variability may reflect differences in patient selection, sampling technique, or thresholds for interpreting results. These results suggest that although BIJVS can provide valuable guidance if there is lateralisation. However, it has significant limitations in the context of a negative result.

Despite this, cure rates following BIJVS-guided surgery were consistently high when reported, ranging from 98% to 100% in studies that provided explicit definitions. This suggests that in appropriately selected patients, BIJVS can contribute to successful surgical outcomes. However, only three studies clearly defined ‘cure’; and several others reported follow-up biochemistry without specifying criteria, limiting the strength of this conclusion.

Notably, one randomized controlled trial (Barczynski et al., 2009) provided higher-quality evidence, demonstrating improved intraoperative localisation accuracy with BIJVS despite equivalent cure rates. Its inclusion strengthens the overall evidence base but also highlights the scarcity of well-designed comparative studies in this field.

A key limitation in the included studies is the heterogeneity in methodology, particularly in lateralisation criteria, reporting of diagnostic metrics, and outcome definitions. Few studies included patients without BIJVS as a comparison group, making it difficult to quantify the added value of BIJVS over conventional imaging or intraoperative judgement. Additionally, most studies lacked long-term follow-up or detailed reporting on whether BIJVS influenced surgical strategy, which could offer insights into its practical utility.

This review also has methodological limitations. Screening was conducted collaboratively rather than independently, and data extraction was performed by one reviewer with another reviewer cross-checking the collected data. This has potential to introduce selection or reporting bias. Only English-language publications were included. Finally, this review did not include a formal risk-of-bias or certainty-of-evidence assessment, in line with the exploratory nature of scoping reviews and the heterogeneity of the included studies [23]. Future systematic reviews with more homogeneous data could apply formal bias-assessment tools to evaluate the methodological quality and strength of evidence for BIJVS.

## Conclusion

Overall, the available evidence suggests that BIJVS can offer high diagnostic yield and contribute to excellent surgical outcomes when used in experienced hands and in selected patients. The existing literature demonstrates that a positive lateralizing result can be clinically informative and often correlates with successful surgical outcomes. However, the lack of standardised protocols, comparative studies, and uniform outcome reporting limits broader generalisability. Future prospective studies are needed to establish clear guidelines for BIJVS use, including standard lateralisation criteria and consistent outcome definitions, to clarify its role in modern parathyroid surgery.

## Data Availability

No datasets were generated or analysed during the current study.

## References

[1] Yeh MW, Ituarte PHG, Zhou HC, Nishimoto S, Amy Liu IL, Harari A et al (2013) Incidence and prevalence of primary hyperparathyroidism in a Racially mixed population. J Clin Endocrinol Metab 98(3):1122–1129 23418315 10.1210/jc.2012-4022 PMC3590475

[2] Bilezikian JP, Brandi ML, Eastell R, Silverberg SJ, Udelsman R, Marcocci C et al (2014) Guidelines for the management of asymptomatic primary hyperparathyroidism: summary statement from the fourth international workshop. J Clin Endocrinol Metab 99(10):3561–3569 25162665 10.1210/jc.2014-1413 PMC5393490

[3] Wilhelm SM, Wang TS, Ruan DT, Lee JA, Asa SL, Duh QY et al (2016) The American association of endocrine surgeons guidelines for definitive management of primary hyperparathyroidism. JAMA Surg 151(10):959 27532368 10.1001/jamasurg.2016.2310

[4] Cheung K, Wang TS, Farrokhyar F, Roman SA, Sosa JA (2012) A Meta-analysis of preoperative localization techniques for patients with primary hyperparathyroidism. Ann Surg Oncol 19(2):577–583 21710322 10.1245/s10434-011-1870-5

[5] Latge A, Riehm S, Vix M, Bani J, Ignat M, Pretet V et al (2021) 18F-Fluorocholine PET and 4D-CT in patients with persistent and recurrent primary hyperparathyroidism. Diagnostics 11(12):2384 34943620 10.3390/diagnostics11122384 PMC8700343

[6] Johnson NA, Tublin ME, Ogilvie JB (2007) Parathyroid imaging: technique and role in the preoperative evaluation of primary hyperparathyroidism. Am J Roentgenol 188(6):1706–1715 17515397 10.2214/AJR.06.0938

[7] Irvin GL, Molinari AS, Figueroa C, Carneiro DM (1999) Improved success rate in reoperative parathyroidectomy with intraoperative PTH assay. Ann Surg 229(6):874 10363902 10.1097/00000658-199906000-00015 PMC1420835

[8] Carneiro-Pla D (2011) Contemporary and practical uses of intraoperative parathyroid hormone monitoring. Endocr Pract 17:44–53 21247846 10.4158/EP10304.RA

[9] Ito F, Sippel R, Lederman J, Chen H (2007) The utility of intraoperative bilateral internal jugular venous sampling with rapid parathyroid hormone testing. Ann Surg 245(6):959–963 17522522 10.1097/01.sla.0000255578.11198.ff PMC1876969

[10] Norman J, Politz D (2009) 5,000 parathyroid operations without frozen section or PTH assays: measuring individual parathyroid gland hormone production in real time. Ann Surg Oncol 16(3):656–666 19130135 10.1245/s10434-008-0276-5

[11] Ibraheem K, Toraih EA, Haddad AB, Farag M, Randolph GW, Kandil E (2018) Selective parathyroid venous sampling in primary hyperparathyroidism: A systematic review and meta-analysis. Laryngoscope 128(11):2662–2667 29756350 10.1002/lary.27213

[12] Tricco AC, Lillie E, Zarin W, O’Brien KK, Colquhoun H, Levac D et al (2018) PRISMA extension for scoping reviews (PRISMA-ScR): checklist and explanation. Ann Intern Med 169(7):467–473 30178033 10.7326/M18-0850

[13] Maceri DR, Kokot N, Green K, Montgomery V, Sharifi J (2011) Split central venous sampling of parathyroid hormone: an adjunct to surgical exploration. Head Neck 33(12):1715–1718 21322077 10.1002/hed.21659

[14] Alvarado R, Meyer-Rochow G, Sywak M, Delbridge L, Sidhu S (2010) Bilateral internal jugular venous sampling for parathyroid hormone determination in patients with nonlocalizing primary hyperparathyroidism. World J Surg 34(6):1299–1303 20372897 10.1007/s00268-010-0556-7

[15] Barczynski M, Konturek A, Hubalewska-Dydejczyk A, Cichon S, Nowak W (2009) Utility of intraoperative bilateral internal jugular venous sampling with rapid parathyroid hormone testing in guiding patients with a negative Sestamibi scan for minimally invasive parathyroidectomy–a randomized controlled trial. Langenbecks Arch Surg 394(5):827–835 19529955 10.1007/s00423-009-0516-6

[16] Sharma J, Milas M, Berber E, Mazzaglia P, Siperstein A, Weber CJ (2008) Value of intraoperative parathyroid hormone monitoring. Ann Surg Oncol 15(2):493–498 18026797 10.1245/s10434-007-9683-2

[17] Lee LS, Canter RJ, Fraker DL (2006) Intraoperative jugular venous sampling AIDS detection of an undescended parathyroid adenoma. World J Surg 30(4):620–623 16555025 10.1007/s00268-005-0238-z

[18] Solorzano CC, Lee TM, Ramirez MC, Carneiro DM, Irvin GL (2005) Surgeon-performed ultrasound improves localization of abnormal parathyroid glands. Am Surg 71(7):557–562 16089118

[19] Taylor J, Fraser W, Banaszkiewicz P, Drury P, Atkins P (1996) Lateralization of parathyroid adenomas by intra-operative parathormone Estimation. J R Coll Surg Edinb 41(3):174–177 8763181

[20] Ribeiro DK, Neves MC, das, Santos R, de Abrahao O (eds) (2025) M Analysis of PTH serum concentration from internal jugular veins of patients with primary hyperparathyroidism. Braz J Otorhinolaryngol 91(1Suppl 1):101605 10.1016/j.bjorl.2025.101605 PMC12013386 40209343

[21] Bergenfelz A, Algotsson L, Roth B, Isaksson A, Tibblin S (1996) Side localization of parathyroid adenomas by simplified intraoperative venous sampling for parathyroid hormone. World J Surg 20(3):358–360 8661845 10.1007/s002689900058

[22] Pattou F, Oudar C, Huglo D, Racadot A, Carnaille B, Proye C (1998) Localization of abnormal parathyroid glands with jugular sampling for parathyroid hormone, and Subtraction scanning with Sestamibi or Tetrofosmine. Aust N Z J Surg 68(2):108–111 9494000 10.1111/j.1445-2197.1998.tb04717.x

[23] Peters MDJ, Marnie C, Colquhoun H, Garritty CM, Hempel S, Horsley T et al (2021) Scoping reviews: reinforcing and advancing the methodology and application. Syst Rev 10(1):263 34625095 10.1186/s13643-021-01821-3 PMC8499488

